# The Vaccination Question

**Published:** 1895-09

**Authors:** 


*The Vaccination Question.* By ARTHUR WOLLASTON HUTTON. Pp. 128. London: METHUEN & CO. 1894.

We have read a great many strange things, and we have heard a great many wild statements; but we do not ever remember before coming across an intelligent man like Mr. Hutton, more assertive of doing strict justice to a subject, and at the same time displaying more prejudice and preconceived ideas. Vaccination is no good—and worse—because the author has determined that it is of no avail! he "will not be learned nor understand, but walks on still in darkness" (p. 70). We do not intend to deal with every point raised in this little work; if we can knock down, as we believe we can, some of his strongholds, the smaller outworks cannot well be held.

Jenner, like most earnest and enthusiastic men, did unquestionably maintain the life efficacy of vaccination. Time has gone on and has shown that vaccination is not more potent against an attack of small-pox than small-pox itself. An attack of severe unmitigated small-pox does not invariably render a person immune to a second attack, or even a second attack to a third; but these cases are very rare, and the supporters of vaccination may admit that small-pox is, perhaps, rather more protective than vaccination and revaccination about the twelfth year: but the process is a very dreaded and dreadful one.

Then there is not a jot of proof that vaccination has any connection with syphilis,[1] as some modern pseudo-scientists assert. McVail has shown conclusively that deaths from infantile syphilis[1] are as frequent in Scotland during the first six months of life as in England; and yet vaccination is not carried out in Scotland until the sixth month and later. Whereas if vaccination were modified syphilis, the roll of deaths in England under six months, ought to outnumber those in Scotland, but they do not; nay more, the averages for both countries continue as nearly as possible alike, through all ages. Then again, we never knew that people who had syphilis, or who had had it, derived any special protection against small-pox in the way that vaccination unquestionably does confer it, which surely ought to be the case were vaccine modified specific germs.

There are many more assertions we should like to confute did space allow, but one we must notice. Certain very careful statistics have been published in Germany, where vaccination and re-vaccination have been rigidly carried out: they show that small-pox mortality is less there than in any other part of Europe, and epidemics are unknown. This cannot be the result of sanitation only. Sanitary measures have never produced such astounding results in other diseases, much as fevers and cholera have been diminished in their incidence. We pass on to deal with Tables I. and II. (pp. 68, 69), upon which the author evidently pins his faith. These tables are set forth to show how twenty-two towns or districts (not including London) have suffered from small-pox, during the last seven-and-a-half years up to June, 1894; and also how much, or how little, they have carried out vaccination; and the author assumes as a fair argument, that where vaccination has been most efficiently done, if there is any good in it, there should be a smaller incidence of small-pox, and, consequently, fewer deaths; and, on the other hand, where vaccination has been most neglected, there small-pox should show up the most disastrously.

In Table I. let us take Bristol, with its (assumed) 5.2 per cent. of vaccination default only, and compare it with Dewsbury with its 37.1 per cent. default. The deaths in Dewsbury are given as 126 (but there are one-and-a-half years short), and in Bristol at 123 (over-stated): 123 in well-vaccinated Bristol. 126 in badly-protected Dewsbury! *Primâ facie*, the latter very little worse off than Bristol! But, what an unfair, dishonest position! Dewsbury has, in round numbers, only a population of 30,000, whilst that of Bristol is 230,000. In a Dewsbury the size of Bristol, the deaths would have been not 126, but at the

[1] *Vaccination Vindicated*, 1887, pp. 136, 138.

very least 126 × 7, or 882! In a Bristol the size of Dewsbury, the deaths would have been 123 ÷ 7, or 17 only.

Take Sheffield: Mr. Hutton states the deaths at 711, in a population of, say, 324,000, or nearly eleven times the size of Dewsbury. In the same ratio as Dewsbury, they would have been 1386. Or, again, to take the boasted Leicester. We will admit that the place has escaped, so far, a severe incidence of small-pox; but what is the mortality of vaccinated persons attacked, as compared with the unvaccinated? 1.1 per cent. in the former, 15.8 per cent. in the latter, according to most trustworthy figures published in the *British Medical Journal*. We may safely predict that Leicester will have its day of reckoning yet: and such a town not only shows a very bad example, but disperses a large number of unvaccinated persons far and wide, and thus inflicts most unfair conditions on other communities. After these statistics and their fallacies, we can appraise at its right value the "reasonable hypothesis of constitutional immunity" (p. 72), as an explanation of the freedom of revaccinated cases from small-pox, and laugh at the charge that vaccination is kept up by doctors in Harley Street (note p. 111), and the corporation of doctors generally, for pecuniary interest. The gravamen is absurd in the extreme. It would be far more profitable for medical men to vaccinate and revaccinate themselves, their families, and friends, and then let small-pox run rampant.

Mr. Hutton is greatly to be blamed for the introduction of the almost blasphemous picture of calf-worship (p. 115), and, being librarian of a large political club, he ought to be specially censured for publishing a book without an index.

